# SARS-CoV-2–Specific Antibodies in Domestic Cats during First COVID-19 Wave, Europe

**DOI:** 10.3201/eid2712.211252

**Published:** 2021-12

**Authors:** Claudia Schulz, Byron Martina, Monica Mirolo, Elisabeth Müller, Ruth Klein, Holger Volk, Herman Egberink, Mariana Gonzalez-Hernandez, Franziska Kaiser, Maren von Köckritz-Blickwede, Albert Osterhaus

**Affiliations:** University of Veterinary Medicine Hannover, Hannover, Germany (C. Schulz, M. Mirolo, H. Volk, M. Gonzalez-Hernandez, F. Kaiser, M. von Köckritz-Blickwede, A. Osterhaus);; Artemis One Health Research Foundation, Delft, the Netherlands (B. Martina, M. Mirolo);; LABOklin, Kissingen, Germany (E. Müller, R. Klein);; Utrecht University Faculty of Veterinary Medicine, Utrecht, the Netherlands (H. Egberink)

**Keywords:** COVID-19, cat, Europe, serology, ELISA, neutralization tests, felines, respiratory infections, severe acute respiratory syndrome coronavirus 2, SARS-CoV-2, SARS, coronavirus disease, One Health, zoonoses, viruses, coronavirus

## Abstract

We conducted a severe acute respiratory syndrome coronavirus 2 antibody seroprevalence study among >2,000 domestic cats from 4 countries during the first coronavirus disease wave in Europe. We found 4.4% seroprevalence using a virus neutralization test and 4.3% using a receptor-binding domain ELISA, demonstrating probable human-to-cat transmission.

Severe acute respiratory syndrome coronavirus 2 (SARS-CoV-2), the cause of the ongoing coronavirus disease (COVID-19) pandemic, causes high rates of illness and death among humans. SARS-CoV-2 is a newly recognized member of the genus *Betacoronavirus*, family *Coronaviridae*, that infects humans. An early serosurvey among domestic cats in Wuhan, China, during January–March 2020 reported 14.7% seropositivity (*1*). Experimental infections demonstrated susceptibility to SARS-CoV-2 infection in cats and other carnivore species, such as ferrets (*Mustela putorius furo*), minks (*Neovison vison*), and to a lesser extent domestic dogs (*2*,*3*), and confirmed anecdotal observations of naturally occurring human-to-animal transmissions (*4*,*5*). Respiratory and gastrointestinal signs were observed in SARS-CoV-2–infected cats (*6*–*8*). We conducted a seroprevalence study for SARS-CoV-2–specific antibodies among domestic cats in Europe during and after the first COVID-19 pandemic wave, using a plaque-reduction virus neutralization test (VNT) and a SARS-CoV-2 receptor-binding domain–specific ELISA (RBD-ELISA).

## The Study

We analyzed serum samples collected from 2,160 domestic cats during April–June 2020. Samples had been sent to a veterinary diagnostic laboratory (LABOklin; Kissingen, Germany) for diagnostic purposes unrelated to suspicion of SARS-CoV-2 infection (*9*). Samples were from 1,136 cats in Germany, 331 in the United Kingdom, 333 in Italy, and 360 in Spain. Among 1,799 samples with demographic data, cats ranged from 0.1–23 years of age (median and mean age 11 years). We estimated a minimum of 300 total samples per location to enable a realistic estimation for each location. To confirm specificity of the assays to detect SARS-CoV-2–specific antibodies, we included 25 prepandemic cat serum samples and 25 serum samples from cats that tested positive for feline coronavirus/feline infectious peritonitis (FCoV/FIP) by NovaTec VetLine (Novatec Immundiagnostica GmbH, https://www.novatec-id.com), a commercial antibody test, in the screening.

We tested all serum samples by VNT, as previously described (*10*). We considered serum samples positive when titers were >20, expressed as the reciprocal of the dilution that gave >80% reduction of stained cells in the plaque reduction neutralization test (PRNT_80_) (Appendix).

We also tested serum samples with an indirect ELISA we developed and validated inhouse. We used an ELISA previously used for detecting SARS-CoV-2 RBD antibodies in human serum (*11*) and replaced the anti-human IgG conjugate with an anti-cat IgG conjugate (Appendix).

We evaluated performance characteristics of the cat ELISA-RBD by using Pearson correlation of the results obtained by ELISA-RBD and Gaussian distribution analyses for the VNT. We also calculated diagnostic sensitivity and specificity of the ELISA-RBD compared with VNT. We conducted data analyses using R (R Foundation for Statistical Computing, https://www.r-project.org) and Prism version 9 (GraphPad Software Inc., https://www.graphpad.com). We calculated SARS-CoV-2 seroprevalence in cats separately for each country.

We found overall SARS-CoV-2 seroprevalence among cats was 4.2% in Germany, 3.3% in the United Kingdom, 4.2% in Italy, and 6.4% in Spain (Table 1; Figure). Among all 2,160 cat serum samples tested, 96 (4.4%, 95% CI 3.6%–5.4%) were positive by VNT and 92 (4.3%, 95% CI 3.4%–5.2%) by RBD-ELISA. The RBD-ELISA showed a diagnostic sensitivity of 90.6% (95% CI 90.0%–91.2%) and specificity of 99.8% (95% CI 99.8%–99.8%) compared with VNT (Table 2). Furthermore, correlation (*r* = 0.9, 95% CI 0.9–0.9) and Gaussian distribution analyses (*r^2^*>0.7) revealed high agreement between VNT and RBD-ELISA sensitivities. All 25 prepandemic serum samples and 25 FCoV/FIP-positive samples tested SARS-CoV-2–negative in both the VNT and RBD-ELISA (data not shown), confirming the specificity of the assay for measuring SARS-CoV-2–specific antibodies.

**Table 1:** Overall VNT SARS-CoV-2 seroprevalence in cats by country during the first pandemic wave, Europe, April–August 2020*

Location	No. tested	No. positive	% Positive (95% CI†)
Germany	1,136	48	4.2 (3.1–5.6)
United Kingdom	331	11	3.3 (1.7–5.9)
Italy	333	14	4.2 (2.3–7.0)
Spain	360	23	6.4 (4.1–9.4)
Total	2,160	96	4.4 (3.6–5.4)

*Seroprevalence determined by virus neutralization test (VNT). Similar results were found with RBD-ELISA, 4.3% (96/2,160; 95% CI 3.6%–5.4%) were seropositive (Table 2). RBD-ELISA, receptor-binding domain–specific ELISA; SARS-CoV-2, severe acute respiratory syndrome coronavirus 2; VNT, virus neutralization test. †Calculated by using 2-sided exact binomial test in R (R Foundation for Statistical Computing, https://www.r-project.org).

**Figure:**
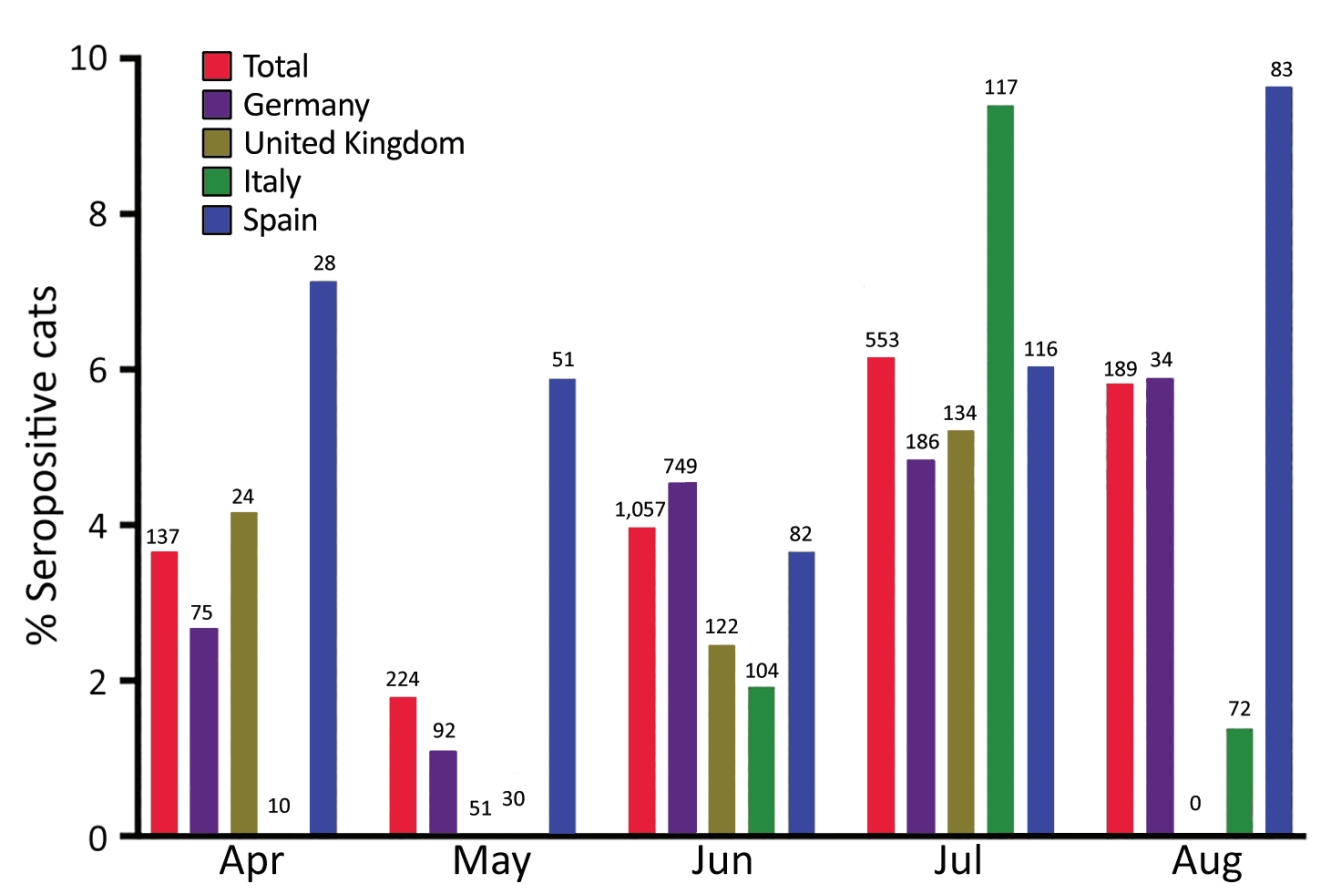
Overall seroprevalence of severe acute respiratory syndrome coronavirus 2 neutralizing antibodies in 2,160 domestic cats, by month and country, during the first coronavirus disease pandemic wave, Europe, April–August 2020. Numerals at the top of each column represent the number of samples collected. Seroprevalence rates peaked in July or August at <9.6% (95% CI 4.25%–18.11%) in Spain (Tables 1, 2).

**Table 2:** Comparison of diagnostic sensitivity and specificity of the RBD-ELISA and VNT in a study of SARS-CoV-2 seroprevalence among domestic cats during the first pandemic wave, Europe, April–August 2020*

Test results	Value
RBD-ELISA sensitivity, % (95% CI)	90.6 (90.0–91.2)
RBD-ELISA specificity, % (95% CI)	99.8 (99.8–99.8)
No. positive (%; 95% CI), n = 2,160	
RBD-ELISA and VNT	87 (4.0; 3.2–4.9)
RBD-ELISA only	92 (4.3; 3.5–5.2)
VNT only	96 (4.4; 3.6–5.4)

*A total of 5 samples were positive with RBD-ELISA and negative with VNT; 9 samples were positive with VNT but negative with RBD-ELISA. RBD-ELISA, receptor-binding domain–specific ELISA; SARS-CoV-2, severe acute respiratory syndrome coronavirus 2; VNT, virus neutralization test.

Our study of domestic cat serum from 4 selected countries showed that during the first COVID-19 wave in Europe, >4% of domestic cats had been infected with SARS-CoV-2, probably through their contacts with infected humans. Because serum samples were sent to the veterinary diagnostic laboratory for conditions unrelated to a suspected SARS-CoV-2 infection, our data might not fully represent the overall seropositivity of the domestic cat population in Europe.

We used a VNT and an RBD-ELISA based on the original SARS-CoV-2 wild-type isolate (Wuhan-Hu-1, GenBank accession no. MN908947.3). The RBD-ELISA proved to have a high sensitivity and specificity compared with the VNT (Table 2), but 5 low-titer (titer = 20) VNT-positive samples remained undetected by the RBD-ELISA. These samples might have remained undetected because of the high specificity of RBD-ELISA, which detects antibodies toward the single spike protein ectodomain. Unlike RBD-ELISA, VNT might identify a broader range of virus neutralizing antibodies, including those directed against other domains of the spike protein. Of note, the only correlation of virus protection we have to date is virus neutralization, which apparently correlates well with RBD-ELISA positivity. For serologic screening and for individual diagnostic testing of domestic cats, the RBD-ELISA could replace the VNT, thus avoiding the use of live SARS-CoV-2 under Biosafety Level 3 laboratory conditions. We further confirmed specificities of the VNT and RBD-ELISA by showing that prepandemic and FCoV/FIP-positive cat serum samples were negative in both assays. This finding excluded the detection of cross-reactive antibodies against feline alphacoronaviruses (*4*) and alphacoronaviruses of other animal species that might infect cats (*4*,*12*). Our data contrast a heavily affected area in China at the onset of the pandemic from which seropositivity levels of domestic cats ranged <15% (*1*), although those results were from relatively fewer tested cats and used a different assay.

## Conclusions

During the first COVID-19 pandemic wave, reported seroprevalence levels in domestic cats ranged from 0.4% in the Netherlands (*4*) to 23% among cats in COVID-19–positive households in France (*13*). Similar seroprevalence levels in cats and humans in the same areas found by us and others suggest that in the absence of another known source (*4*,*13*; C. Schulz, unpublished data) (Appendix Table), SARS-CoV-2 infections in cats are most likely due to human-to-cat contact transmission.

Most natural SARS-CoV-2 infections of cats appear to run a mild or subclinical course, with respiratory or gastrointestinal clinical signs reported in confirmed natural infections (*6*–*8*). Evidence from experimental studies suggests that cats are susceptible to SARS-CoV-2 infection and can maintain the virus within a cat population and spill the infection backward or forward to other species (*2*,*3*,*14*). However, no evidence of cat-to-human transmission, nor of cat-specific mutations or variants of SARS-CoV-2, has been detected thus far (*8*,*12*,*15*). This finding contrasts reports on minks kept in farms, where mink-to-human spillback infections and mink-specific mutations have been reported (*5*). Although no evidence currently suggests that domestic cats play a role in the epidemiology of human SARS-CoV-2 infection, clinicians and veterinary practitioners should recommend that SARS-CoV-2–infected persons avoid close contact with their domestic cats and practice the same nonpharmaceutical prevention measures toward cats as they do to prevent human-to-human infection.

Appendix Additional information on SARS-CoV-2 seropositivity in domestic cats during the first COVID-19 wave, Europe.
