# Supplementary material for: SARS-CoV-2–Specific Antibodies in Domestic Cats during First COVID-19 Wave, Europe
Source: Emerg Infect Dis. 2021 Dec;27(12):3115–8. doi: 10.3201/eid2712.211252 (PMC8632155; doi:10.3201/eid2712.211252)
Supplement: Appendix — Additional information on SARS-CoV-2 seropositivity in domestic cats during the first COVID-19 wave, Europe. [file 21-1252-Techapp-s1.pdf]

Article DOI: https://doi.org/10.3201/eid2712.211252

SARS-CoV-2–Specific Antibodies in Domestic Cats during First COVID-19 Wave, Europe

Appendix

Virus Neutralization Test

We used a plaque reduction virus neutralization test (VNT) to detect severe acute respiratory syndrome coronavirus 2 (SARS-CoV-2) in cat serum samples as follows. We performed a 2-fold serial dilution of 1:20 diluted serum in culture medium and incubated with 500 50% tissue culture infectious dose ($TCID_{50}$) of SARS-CoV-2 strain Wuhan-Hu-1 for 1 h. Then we added the mixture to Vero cells and incubated for 1 h, after which we washed the cells and incubated in medium for another 8 h. We then fixed the cells with 4% paraformaldehyde and stained by using a rabbit anti–SARS-CoV serum (Sino Biological, https://www.sinobiological.com) and a secondary Alexa Fluor488–labeled goat anti-rabbit IgG (Invitrogen, https://www.thermofisher.com). We counted the number of infected cells per well by using the Cytation 1 imager (Biocompare, https://www.biocompare.com). Serum samples with titers >20, expressed as the reciprocal of the dilution that gave that gave >80% reduction of stained cells in the plaque reduction neutralization test ($PRNT_{80}$), were considered positive.

Receptor Binding Domain–Specific ELISA

We produced a SARS-CoV-2 receptor binding domain–specific ELISA (RBD-ELISA), as previously described (*1*). In brief, the RBD of spike 1 protein (S1) of SARS-CoV-2 residues belonging to strain SARS-CoV-2 Wuhan-Hu-1 were expressed through the expression plasmid pCAGGS in human embryonic kidney 293 cells (HEK-293T) cells. Constructs carried a C-terminal trimerization motif to induce RBD polymerization and a Strep-tag detection protein to facilitate RBD protein purification. In brief, we coated Costar plates (Corning Inc., https://www.corning.com) with 1 μg/mL of purified SARS-CoV-2 RBD antigen in 1%

phosphate-buffered saline (PBS) overnight at 4°C. Subsequently, we blocked plates with 1% skimmed milk powder (Campina, https://www.campina.nl) in 1% PBS and incubated for 1 h at 37°C. After blocking, we added 100 μL of 1:50 cat serum in blocking buffer containing 0.05% Tween-20, and 3% NaCl to each microplate well.

Controls on each plate included blank/conjugate only control, known positive cat serum controls, and archival serum negative control. We diluted secondary antibody, HRPO-labeled anti-cat (Rockland Immunochemicals, Inc., https://rockland-inc.com) at 1:10,000 in dilution buffer and then added the conjugate to each well and incubated for 1 h at 37°C. We filled each microwell with 100 μL of TMB-ELISA Substrate Solution (Thermo Fisher Scientific, https://www.thermofisher.com) for 10 min before stopping the reaction with same volume of 2 mol Alkaline Phosphatase Stop Solution (Sigma-Aldrich, https://www.sigmaaldrich.com). We read plates for optical density (OD) at 450 and 650 nm by using an RT-2100C Microplate Reader (Rayto Life and Analytical Sciences, https://www.rayto.com) within 30 min of stopping the reaction. We calculated the OD as the absorbance at 450 nm minus the OD at 650 nm to remove background noise before statistical analysis.

We set a change-point method according to available literature to establish the ELISA reliability and distinction between positive and negative cat serum samples for SARS-CoV-2 antibodies (16S). We determined optimal conditions to test the specificity of the RBD-ELISA. At a 1:50 serum dilution in RBD-ELISA, we observed an acceptable amount of background signal from the negative controls; at this dilution, the OD value of the positive control cat serum was ≥6 times the OD value of the negative control. Based on these data, we used a threshold of 3 SD above the mean of the negative controls for the RBD-ELISA. We considered serum samples with an OD value ≥0.1 nm positive for SARS-CoV-2 and those with an OD value <0.1 nm negative. We calculated the diagnostic sensitivity and specificity of the RBD-ELISA compared with VNT, as described previously (*2*).

References

1. Okba NMA, Müller MA, Li W, Wang C, GeurtsvanKessel CH, Corman VM, et al. Severe acute respiratory syndrome coronavirus 2–specific antibody responses in coronavirus disease patients. Emerg Infect Dis. 2020;26:1478–88. PubMed https://doi.org/10.3201/eid2607.200841

2. Jendrny P, Schulz C, Twele F, Meller S, von Köckritz-Blickwede M, Osterhaus ADME, et al. Scent dog identification of samples from COVID-19 patients–a pilot study. BMC Infect Dis. 2020;20:536. PubMed https://doi.org/10.1186/s12879-020-05281-3

3. Fischer B, Knabbe C, Vollmer T. SARS-CoV-2 IgG seroprevalence in blood donors located in three different federal states, Germany, March to June 2020. Euro Surveill. 2020;25:1–4. PubMed https://doi.org/10.2807/1560-7917.ES.2020.25.28.2001285

4. Michelitsch A, Hoffmann D, Wernike K, Beer M. Occurrence of antibodies against SARS-CoV-2 in the domestic cat population of Germany. Vaccines (Basel). 2020;8:1–10. PubMed https://doi.org/10.3390/vaccines8040772

5. Signorelli C, Zucchi A, Tersalvi CA, Ciampichini R, Beato E, Balzarini F, et al. High seroprevalence of SARS_COV-2 in Bergamo: evidence for herd immunity or reason to be cautious? Int J Public Health. 2020;65:1815–7. PubMed https://doi.org/10.1007/s00038-020-01524-x

6. Bassi F, Arbia G, Falorsi PD. Observed and estimated prevalence of Covid-19 in Italy: How to estimate the total cases from medical swabs data. Sci Total Environ. 2021;764:142799. PubMed https://doi.org/10.1016/j.scitotenv.2020.142799

7. Cito F, Amato L, Di Giuseppe A, Danzetta ML, Iannetti S, Petrini A, et al. A covid-19 hotspot area: activities and epidemiological findings. Microorganisms. 2020;8:1711. PubMed https://doi.org/10.3390/microorganisms8111711

8. Patterson EI, Elia G, Grassi A, Giordano A, Desario C, Medardo M, et al. Evidence of exposure to SARS-CoV-2 in cats and dogs from households in Italy. Nat Commun. 2020;11:6231. PubMed https://doi.org/10.1038/s41467-020-20097-0

9. Public Health England. Sero-surveillance of COVID-19 [cited 2021 Jan 23]. https://www.gov.uk/government/publications/national-covid-19-surveillance-reports/sero-surveillance-of-covid-19

10. Pollán M, Pérez-Gómez B, Pastor-Barriuso R, Oteo J, Hernán MA, Pérez-Olmeda M, et al.; ENE-COVID Study Group. Prevalence of SARS-CoV-2 in Spain (ENE-COVID): a nationwide, population-based seroepidemiological study. Lancet. 2020;396:535–44. PubMed https://doi.org/10.1016/S0140-6736(20)31483-5

11. Fritz M, Rosolen B, Krafft E, Becquart P, Elguero E, Vratskikh O, et al. High prevalence of SARS-CoV-2 antibodies in pets from COVID-19+ households. One Health. 2021;11:100192. PubMed https://doi.org/10.1016/j.onehlt.2020.100192

12. Slot E, Hogema BM, Reusken CBEM, Reimerink JH, Molier M, Karregat JHM, et al. Low SARS-CoV-2 seroprevalence in blood donors in the early COVID-19 epidemic in the Netherlands. Nat Commun. 2020;11:5744. PubMed https://doi.org/10.1038/s41467-020-19481-7

13. Vos ERA, den Hartog G, Schepp RM, Kaaijk P, van Vliet J, Helm K, et al. Nationwide seroprevalence of SARS-CoV-2 and identification of risk factors in the general population of the Netherlands during the first epidemic wave. J Epidemiol Community Health. 2020;75:489–95. PubMed https://doi.org/10.1136/jech-2020-215678

14. Zhao S, Li W, Schuurman N, van Kuppeveld F, Bosch B-J, Egberink H. Serological screening for coronavirus infections in cats. Viruses. 2019;11:743. PubMed https://doi.org/10.3390/v11080743

Appendix Table. Seroprevalence in humans and cats by selected country during the first wave of the COVID-19 pandemic in Europe, 2020*

	Human		Cat (our study)		Reference	
Country	Seroprevalence	Month	Seroprevalence	Month	Human	Cat
Germany	0.9–2.2†	Mar–Jun†	0.7 (4.2)	Apr–Sep (Apr–Aug)	Fischer et al. 2020 (*3*)	Michelitsch et al. 2020 (*4*)
Italy, northern Italy	9 to 42%	Feb–Jul	3.9 (4.2)	Mar–May (Apr–Aug)	Signorelli et al. 2020 (*5*), Bassi et al. 2020 (*6*), Cito et al. 2020 (*7*)	Patterson et al. 2020 (*8*)
United Kingdom, Midlands	7%	Apr	– (4.2/5.8)	– (Apr/Apr-Aug)	Public Health England (*9*)	
Spain	5.0	Apr–May	–	–	Pollán et al. 2020 (*10*)	
Spain, Madrid	>10.0	Apr–May	– (6.3/6.4)	– (Apr–May/Apr–Aug)	Pollán et al. 2020 (*10*	
France, COVID-19–positive households, Besancon	100	Jun	23.5 (–)	Jun (–)		Fritz et al. 2020 (*11*)
France, COVID-19 unknown status of households, Besancon	–	–	6.3 (–)	Jun (–)		Fritz et al. 2020 (*11*)
The Netherlands	2.7–9.5	April–May	0.4 (–)	April–May (–)	Slot et al. 2020 (*12*) (*13*), Vos et al. 2020	Zhao et al. 2021 (*14*)

*COVID-19, coronavirus disease; –, no data available.
†Low-incidence federal states of North-Rhine Westphalia, Lower Saxony, and Hesse.
